# Intensive Frequent Granulocyte Adsorptive Apheresis Therapy for Acute Fulminant Ulcerative Colitis: Two Consecutive Case Reports

**DOI:** 10.7759/cureus.43599

**Published:** 2023-08-16

**Authors:** Yoshitaka Furuto, Rikimaru Sawada, Akio Namikawa, Nobuyuki Matsuhashi, Yuko Shibuya

**Affiliations:** 1 Department of Hypertension and Nephrology, NTT Medical Centre Tokyo, Tokyo, JPN; 2 Department of Gastrointestinal Endoscopy, NTT Medical Centre Tokyo, Tokyo, JPN

**Keywords:** intensive treatment, disease severity, rapid remission, granulocyte/monocyte adsorptive apheresis, apheresis, ulcerative colitis

## Abstract

Granulocyte/monocyte adsorptive apheresis (GMA) therapy is a treatment method for ulcerative colitis (UC). Twice-weekly GMA regimens are usually administered to treat severe UC. Although GMA efficacy is considered frequency-dependent, there is no uniformly accepted optimal GMA regimen, and there is insufficient evidence regarding optimal GMA therapy frequency for acute fulminant UC. Case 1 was of a 33-year-old man, and case 2 was of a 20-year-old woman. They were diagnosed with acute fulminant UC and treated with steroid therapy, but exhibited exacerbated UC, and their conditions worsened. We, therefore, initiated intensive frequent GMA therapy (conducted 10-11 times during a 13-day period). In both cases, remission was achieved within two weeks of therapy induction. Herein, we describe two consecutive cases in which rapid remission of acute fulminant UC was achieved without adverse events using intensive frequent GMA therapy. These cases suggest that intensive frequent GMA therapy might induce rapid remission in acute fulminant UC cases and may be more effective than twice-weekly GMA regimens.

## Introduction

Ulcerative colitis (UC) is an intractable inflammatory bowel disease of unknown cause, which exhibits a pattern of repeated relapse and remission. Combinations of 5-aminosalicylic acid, steroids, immunosuppressants, and biological preparations can induce remission in many UC cases; however, adverse events are common with these drugs. Meanwhile, severe UC is often associated with granulocyte and monocyte infiltration in the bowel mucosa. Granulocyte/monocyte adsorptive apheresis (GMA) therapy is a treatment method developed in Japan that uses an extracorporeal circulation device to adsorb and remove WBCs from the patient’s peripheral blood, thereby suppressing immune reactions and reducing inflammation. This therapy is characterized by fewer side effects and greater effectiveness than steroids [1,2]. Moreover, the efficacy and safety of GMA have been demonstrated in older and pregnant patients with UC [3]. Herein, we describe two consecutive cases of acute fulminant UC, wherein rapid remission was achieved with intensive frequent GMA therapy administered 10-11 times within two weeks.

## Case presentation

This study was conducted according to the guidelines of the Declaration of Helsinki. It was approved by the Institutional Ethics Committee of NTT Medical Centre, Tokyo. Written informed consent was obtained from the patients for the publication of this case report. The diagnostic criteria for UC according to Japanese guidelines [4] are classified based on clinical severity as follows: (1) number of defecations: six times/day or more; (2) bloody stools: mostly blood; (3) fever: temperature of 37.5°C or more; (4) tachycardia: 90 beats/min or more; (5) anemia: Hb 10 g/day dL or less; 6) erythrocyte sedimentation rate: 30 mm/hour or more or C-reactive protein (CRP): 3.0 mg/dL or more. Severe UC is defined as a case that satisfies either (3) or (4), which are systemic symptoms, in addition to (1) and (2), and satisfies four or more of the six items. Furthermore, among severe cases, those exhibiting particularly intense symptoms are categorized as having a fulminant presentation, and, according to the course of onset, they are divided into acute fulminant type and relapsing fulminant type. The diagnostic criteria for fulminant UC are cases that meet all of the following five criteria: (1) meeting the severity criteria; (2) experiencing bloody diarrhea more than 15 times/day; (3) persistent fever of 38°C or higher; (4) leukocytosis of 10,000/mm3 or more; (5) severe abdominal pain. We applied these diagnostic criteria in the present study, and two cases had an acute onset and met the diagnostic criteria for acute fulminant type. Moreover, we used the Lichtiger index (LI) [5] and the simple clinical colitis activity index (SCCAI) [6] as disease activity evaluation indices for UC and followed up the course of treatment (Table 1). The LI defines severe as ≥11 and remission as ≤3. The SCCAI defines severe as ≥12 and remission as ≤2.

**Table 1:** Laboratory data on admission, pre-GMA, and post-GMA. GMA: Granulocyte/monocyte adsorptive apheresis; WBC: White blood cells; Hb: Hemoglobin; Ht: hematocrit; Alb: albumin; CRP: C-reactive protein, LI: Lichtiger Index (5), SCCAI: simple clinical colitis activity index (6).

	Case 1			Case 2		
	On admission	Pre-GMA	Post-GMA	On admission	Pre-GMA	Post-GMA
Day	1	9	23	1	14	28
WBC (/μL)	9,800	11,800	6,700	9,000	14,500	7,400
Granulocytes	8,030	11,033	4,500	7,020	11,310	5,300
Lymphocytes	850	531	1,790	1,180	1,523	1,400
Monocytes	813	118	308	693	1,305	548
Hb (g/dL)	12.7	10.3	11.1	7.8	7.9	9.4
Ht (%)	39.0	33.4	35.2	24.3	25.2	29.1
Platelets (×10^4^/µL)	34.8	43.0	31.3	39.4	42.4	36
Alb (g/dL)	2.3	2.0	3.2	3.0	2.2	2.3
CRP (mg/dL)	12.2	5.9	<0.3	5.6	6.4	0.5
LI (4)	13	16	1	13	16	3
SCCAI (5)	13	14	1	12	13	2

Case 1

**Figure 1:**
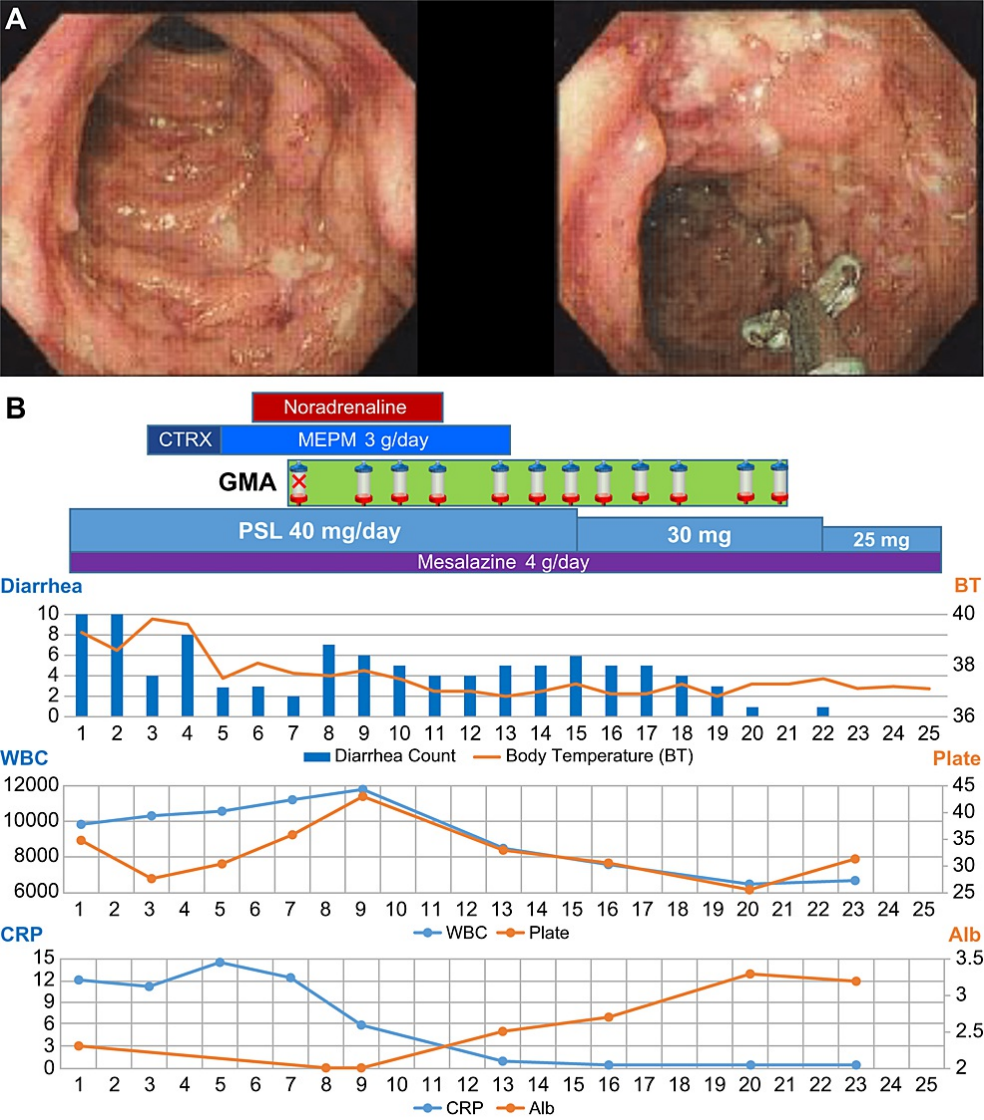
(A) Colonoscopy performed in the previous clinic in Case 1 showing longitudinal ulcers from the ascending to the rectal colon. (B) Case 1 treatment course from day 1 to day 25 following admission. The upper part of the figure displays treatment details. The first GMA session failed because blood access in peripheral blood vessels could not be secured. The middle graphs show diarrhea counts (times/day) and body temperature (℃). The lower graphs show white blood cell count (WBC, counts/µL), platelet count (plate, counts/µL), C-reactive protein level (CRP, mg/dL), and serum albumin level (Alb, g/dL). CTRX, ceftriaxone; MEPM, meropenem; GMA, granulocyte/monocyte adsorptive apheresis; PSL, prednisolone.

The patient, a 33-year-old man, had previously consulted a physician due to symptoms of bloody diarrhea that had persisted for two months. A colonoscopy (Figure 1A) performed two weeks prior to admission revealed longitudinal ulcers from the ascending to the rectal colon. Histopathological examination of the mucous membrane from the ascending to the rectal colon showed diffuse inflammatory cell infiltration and crypt abscesses in all layers, and the patient was diagnosed with pancolitis. The patient was treated with prednisolone (PSL) of 20 mg/day, mesalazine of 4 g/day, and budesonide enema of 2 mg twice/day for two weeks. However, the bloody diarrhea did not improve and persisted for more than 15 times/day. Therefore, the patient was referred and admitted to the gastrointestinal endoscopy department of our hospital. The physical findings upon admission were as follows: body mass index (BMI) of 19.4 kg/m2; blood pressure of 133/67 mmHg; pulse rate of 119/min; body temperature of 39.3°C; percutaneous oxygen saturation (SpO2) of 97% (room air); and clear consciousness. The patient had a numerical rating scale (NRS) pain score of five for abdominal pain, with no muscle defense and no rebound tenderness. He had bloody diarrhea every hour, decreased skin turgor, and had collapsed superficial veins. The patient exhibited a high WBC count of 9,800/µL, and a CRP level of 12 mg/dL. Computed tomography (CT) showed characteristic UC findings, such as intestinal wall thickening, disappearance of haustra, and lead pipe-like changes in the entire colon. Differential diagnoses were excluded as the patient tested negative for cytomegalovirus antigen, his blood culture was negative, and his stool culture was commensal. The patient met the “severe” and “fulminant” criteria of the clinical severity classification for UC [4-6] and was diagnosed as having acute fulminant UC. Treatment with fasting and fluids, 40 mg/day PSL, and 4 g/day mesalazine was initiated; however, the patient’s fever persisted. On the fifth day following admission, his blood pressure dropped to 65/49 mmHg, and he exhibited signs of shock. Subsequently, the transfusion volume was increased, and vasopressor and broad-spectrum antibiotic treatments were started for suspected sepsis. Blood cultures were subsequently found to be negative, and the shock was thought to be caused by decreased circulating plasma volume owing to exacerbation of UC. By the seventh day following admission, serum albumin was 2.0 g/dL, his general condition had worsened, and a chest radiograph revealed bilateral pleural effusion. The gastrointestinal endoscopy department consulted our hypertension and nephrology department for GMA therapy. We attempted GMA therapy; however, securing a blood vessel to draw blood using a 17 G needle proved challenging due to venous collapse, and therapy could not be administered. On the ninth day, a dialysis catheter was inserted into the internal jugular vein, after which GMA therapy was commenced (using an Adacolumn (JIMRO, Takasaki, Japan) membrane; blood flow (Qb), 40 mL/min; blood processing volume, 2,400 mL/round). There were no adverse events from the GMA therapy, and, based on disease severity, infection, and thrombosis risk, intensive frequent GMA therapy, administered 11 times in a 13-day period (on every day except Sunday) was proposed. In universal practice, mesalazine is not recommended for managing acute fulminant ulcerative colitis, and consideration of biologics was warranted. However, we first expected the effect of GMA therapy after sepsis to be differentiated and wanted to avoid the complexities of interpreting the effect of concurrent drug changes. On the 11th day following admission, vasopressor administration was stopped, and elemental nutrition administration was started. On day 13, the fever subsided, and antibiotic administration was stopped. On day 14, a low-residue diet was started, after which the frequency of diarrhea decreased. On day 15, PSL was reduced to 30 mg/day. On day 21, all 11 GMA sessions were completed. On day 22, the patient’s stool quality improved, and he achieved remission with LI, SCCAI [5,6]. The PSL dosage was reduced to 25 mg/day, with symptoms and laboratory, LI, and SCCAI results improving rapidly after the introduction of intensive frequent GMA (Table 1). On day 25, the patient was discharged from hospital. The treatment course, in this case, is shown in Figure 1B.

Case 2

**Figure 2:**
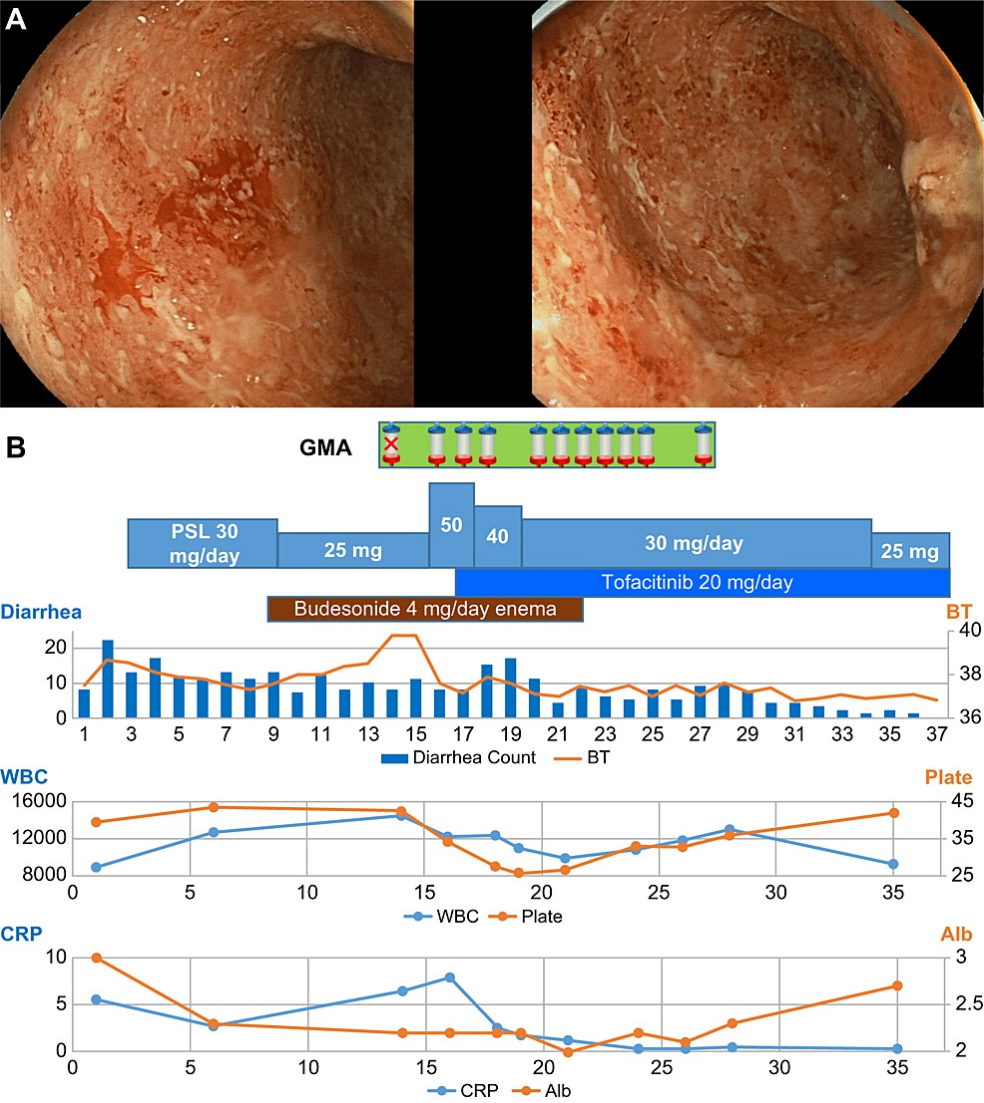
(A) Colonoscopy performed before the treatment course in Case 2 showing continuous rough mucosal lesions and loss of the visible vascular pattern in the sigmoid colon and rectum. (B) Case 2 treatment course from day 1 to day 37 following admission. The upper part of the figure displays treatment details. The first GMA failed because blood access in peripheral blood vessels could not be secured. The middle graphs show diarrhea counts (times/day) and body temperature (℃). The lower graphs show white blood cell count (WBC, counts/µL), platelet count (plate, counts/µL), C-reactive protein level (CRP, mg/dL), and serum albumin level (Alb, g/dL). GMA, granulocyte/monocyte adsorptive apheresis; PSL, prednisolone.

The patient, a 20-year-old woman, presented to the gastrointestinal endoscopy department of our hospital with bloody diarrhea and abdominal pain that persisted for six months. CT showed wall thickening in the sigmoid colon, and colonoscopy (Figure 2A) revealed continuous rough mucosal lesions, as well as loss of visible vascular pattern in the sigmoid colon and rectum. Histopathological examination of the large intestine showed inflammatory cell infiltration into the mucosal interstitium and crypt abscesses, leading to a diagnosis of left-sided UC. The patient had a family history of paternal UC. She received 4.8 g/day oral mesalazine and 1 g/day mesalazine enemas, but the frequency of bloody diarrhea increased, and a fever of 38°C was observed; hence, the patient was hospitalized. The physical findings upon admission were as follows: BMI, 20.8 kg/m2; blood pressure, 95/55 mmHg; pulse rate, 103/min; body temperature, 37.4°C; SpO2, 97% (room air); and consciousness, clear. She had an NRS abdominal pain score of three, with no symptoms of peritoneal irritation. The patient experienced bloody diarrhea about 20 times/day, had decreased skin turgor, and exhibited collapsed superficial blood vessels. The patient had a high WBC count of 9,000/µL, and the CRP level was 5.6 mg/dL. On the first day following admission, the patient started fasting and fluid replacement therapy. Mesalazine was discontinued due to possible symptom exacerbation. On the third day, 30 mg/day PSL was started. On the ninth day, a 2-mg twice/day enema of budesonide was started, but, on the 12th day, the patient developed a fever of 39°C, and abdominal pain worsened. She experienced anemia associated with bloody diarrhea and received blood transfusions as needed (four units, administered on days six, 15, 16, and 26). Differential diagnoses were excluded as the patient was negative for cytomegalovirus antigen, her blood culture was negative, and her stool culture was commensal. She met the “severe” and “fulminant” criteria of the clinical severity classification for UC [4-6] and was diagnosed with acute fulminant UC. On day 14, the gastrointestinal endoscopy department consulted our department regarding GMA therapy, which was attempted; however, it could a blood vessel could not be secured using a 17 G needle due to venous collapse. On day 16, PSL was increased to 50 mg/day. Moreover, a dialysis catheter was inserted into the internal jugular vein, after which GMA therapy was initiated (using an Adacolumn (JIMRO, Takasaki, Japan) membrane; Qb, 40 mL/min; blood processing volume, 2,400 mL/round). There were no adverse events from GMA therapy, and the patient began recovering from the fever. Based on disease severity, infection, and thrombosis risk, intensive frequent GMA therapy (administered 11 times in a 13-day period (every day, except Sunday) was proposed. On day 17, treatment with 20 mg/day tofacitinib was started. Thereafter, the patient’s bloody diarrhea and abdominal pain began to subside, and PSL was tapered. On day 18, central parenteral nutrition was started, followed by elemental nutrition on day 21. On day 27, all 11 GMA sessions were completed (10 sessions were completed in practice because the initial session could not be conducted). On day 28, remission was achieved with LI, SCCAI [5,6], and the patient was switched to a low-residue diet. She was gradually shifted to a normal diet once her stool condition had improved and bloody diarrhea had stopped, with the number of defecations recovering to one to two times/day. The patient’s symptoms and the laboratory LI and SCCAI results improved rapidly after the introduction of intensive frequent GMA (Table 1), and, on day 37, the patient was discharged from the hospital, with 25 mg/day PSL and 20 g/day tofacitinib. The treatment course, in this case, is shown in Figure 2B.

## Discussion

Here, we present two consecutive cases of acute fulminant UC characterized by a persistent exacerbation of symptoms even after steroid administration. The patients’ conditions were not absolute surgical indications for colonic perforation or toxic megacolon. Moreover, both patients wanted to avoid colectomy and preferred medical treatment before the colectomy. Therefore, we carefully monitored their conditions and abdominal findings so as not to miss the timing of the operation while performing systemic management and GMA therapy and implementing other medical treatments. Finally, our intensive and frequent GMA therapy regimen yielded rapid remission within merely two weeks [5,6] and was therefore considered successful (Table 1). To our knowledge, to date, there have been no reports on intensive frequent GMA therapy conducted 10-11 times within 2 weeks. Therefore, this is the first case report regarding short-term frequent GMA. The mechanism by which GMA improves UC involves selective adsorption of granulocytes and monocytes within the column, decreased leukocyte-endothelial adhesion molecule-1, decreased chemotaxis, and induction of qualitative changes in leukocytes and elimination of inflammatory cytokines by increasing soluble tumor necrosis factor α receptors [7,8]. In Japan, GMA therapy has been approved as a treatment option for severe UC by the Japan Ministry of Health and Welfare since April 2000 [1]. Initially, GMA therapy was administered once a week under national insurance coverage in Japan. A subsequent large-scale, multicenter randomized controlled trial [9,10] showed that twice-weekly administration of GMA is more effective than once-weekly administration, as the once-weekly treatment group exhibited a remission induction rate of 54% and a mean remission induction period of 28.1±16.9 days, whereas the twice-weekly treatment group exhibited significantly better results, with a remission induction rate of 71.4% and a mean remission induction period of 14.9±9.5 days [10]. At our facility, although twice-weekly GMA therapy has led to symptomatic improvement in many patients, in some cases of severe or fulminant UC conventional twice-weekly treatment is not effective. There is no uniformly accepted optimal GMA regimen [11], with insufficient evidence regarding optimal GMA therapy frequency for especially fulminant UC [12], indicating the need for GMA therapy optimization. The effectiveness of GMA has been shown to be frequency-dependent, with five consecutive days of GMA therapy for active UC with moderately and severely significantly decreasing CRP without decreasing granulocyte and/or monocyte levels or other serious adverse events and achieving a symptom improvement rate of 70% in the short term [13]. Additionally, a study on the amount of blood processed per GMA therapy session reported that a larger amount of processed blood was more effective for improving symptoms and inducing remission than the regular amount of processed blood, suggesting that GMA efficacy also depends on the amount of processed blood. This suggests that GMA efficacy is dose-dependent, without a concurrent dose-dependent increase in adverse events, such as neutropenia and monocyte depletion [14,15]. The once-weekly GMA therapy protocol was discontinued in April 2010 because of the frequency-dependent response, and currently, GMA is usually administered one to two times/week, with twice-weekly intensive GMA regimens being applied in cases of severe UC [13]. It was also observed that an improved remission rate is associated with the number of GMA cycles (54.0% vs 71.2%, p=0.029 in five-cycle and ten-cycle regimens, respectively) [10]. In Japan, continuous daily GMA therapy is possible under national insurance coverage [15] and generally includes up to 10 sessions of GMA therapy (11 sessions for fulminant UC cases), which are approved as induction therapy. In the Japanese UC guidelines [4], GMA therapy is a treatment option for steroid-resistant and steroid-dependent UC that does not improve after approximately one week of steroid therapy. For severe cases, guidelines suggest implementing GMA therapy at an early stage. However, the optimal frequency of GMA sessions in the treatment regimen for acute fulminant UC during a given time course remains to be established. Evidence regarding the remission induction rate or remission induction period for acute fulminant UC by GMA therapy is limited. GMA requires securing a peripheral vein and conducting extracorporeal circulation with blood removal at 30-40 mL/min. However, in clinical practice, problems are often encountered in acute fulminant UC cases, such as difficulty in securing access to peripheral blood vessels for blood withdrawal due to intractable diarrhea-induced hypoalbuminemia and anemia. In such cases, GMA may be administered through an indwelling dialysis catheter in a central vein. However, catheter placement during UC or steroid administration carries a high risk of thrombosis and catheter infection [16], making long-term catheter placement undesirable and early removal important. In the present cases, we considered intensive frequent GMA therapy reasonable based on the reported efficacy of five consecutive days of GMA therapy [13] and the desire to avoid catheter complications. One report indicated that the hospitalization duration in cases of non-fulminant UC receiving steroid and GMA therapy was 38.7±5.8 days for once-weekly GMA therapy and 22.3±4.1 days for twice-weekly therapy [17]. After the introduction of intensive frequent GMA therapy in the present cases, the patient’s symptoms improved rapidly, and remission was achieved after 10-11 sessions of GMA therapy. The patients in Cases 1 and 2 were discharged after 16 and 22 days, respectively, indicating the effectiveness of the therapy. Understanding the modality of this treatment necessitates more extensive studies.

Some reports have indicated that GMA therapy may be promising in cases with a short UC affliction duration, cases in which GMA therapy was rapidly introduced, and cases where WBC counts decreased after the first session of GMA therapy [18,19]. A recent systematic review and meta-analysis indicated that the addition of adjunctive GMA therapy is more effective than conventional therapy alone in terms of inducing and maintaining remission in patients with UC [11]. This report also indicated that GMA therapy could be cost-effective in the long term, as it may reduce the cost of long-term medical services, such as hospitalization and surgery [11]. Moreover, recent studies have suggested that GMA therapy may be beneficial in patients who no longer respond to biologicals [20]. In Japan, GMA maintenance therapy once every two weeks was included under the national insurance coverage in 2022. Therefore, it is possible to combine intensive frequent GMA therapy for remission induction with subsequent maintenance GMA therapy for cases of acute fulminant UC.

A limitation of the present study is that endoscopic examination findings following the GMA therapy course are not available, as endoscopy was not performed after the therapy because the patient’s symptoms improved remarkably.

## Conclusions

Our experience in treating cases of acute fulminant UC suggests that intensive frequent GMA therapy, administered 10-11 times in a two-week period, may induce more rapid remission than twice-weekly GMA regimens. We emphasize that, in cases of acute fulminant UC, more frequent GMA sessions per week should be flexibly considered to achieve rapid remission or early discharge, rather than adhering strictly to twice-weekly GMA regimens. Further research to establish optimal GMA treatment regimens for UC is required.

## References

[1] Yamamoto T, Saniabadi AR, Maruyama Y, Umegae S, Matsumoto K (2007). Factors affecting clinical and endoscopic efficacies of selective leucocytapheresis for ulcerative colitis. Dig Liver Dis.

[2] Saniabadi AR, Hanai H, Takeuchi K (2003). Adacolumn, an adsorptive carrier based granulocyte and monocyte apheresis device for the treatment of inflammatory and refractory diseases associated with leukocytes. Ther Apher Dial.

[3] Yanagisawa K, Murakami M, Kondo Y (2019). Efficacy and safety of adsorptive granulocyte and monocyte apheresis in elderly and pregnant patients with ulcerative colitis. Ther Apher Dial.

[4] Hisamatsu group; 2023 (2020). Ulcerative colitis/Crohn’s disease diagnostic criteria/treatment guidelines revised edition. http://www.ibdjapan.org/pdf/doc15.pdf.

[5] Lichtiger S, Present DH, Kornbluth A (1994). Cyclosporine in severe ulcerative colitis refractory to steroid therapy. N Engl J Med.

[6] Walmsley RS, Ayres RC, Pounder RE, Allan RN (1998). A simple clinical colitis activity index. Gut.

[7] Takeda Y, Shiobara N, Saniabadi AR, Adachi M, Hiraishi K (2004). Adhesion dependent release of hepatocyte growth factor and interleukin-1 receptor antagonist from human blood granulocytes and monocytes: evidence for the involvement of plasma IgG, complement C3 and beta2 integrin. Inflamm Res.

[8] Takeda Y, Hiraishi K, Takeda H (2003). Cellulose acetate beads induce release of interleukin-1 receptor antagonist, but not tumour necrosis factor-alpha or interleukin-1beta in human peripheral blood. Inflamm Res.

[9] Sakuraba A, Sato T, Naganuma M (2008). A pilot open-labeled prospective randomized study between weekly and intensive treatment of granulocyte and monocyte adsorption apheresis for active ulcerative colitis. J Gastroenterol.

[10] Sakuraba A, Motoya S, Watanabe K (2009). An open-label prospective randomized multicenter study shows very rapid remission of ulcerative colitis by intensive granulocyte and monocyte adsorptive apheresis as compared with routine weekly treatment. Am J Gastroenterol.

[11] Kiss S, Németh D, Hegyi P (2021). Granulocyte and monocyte apheresis as an adjunctive therapy to induce and maintain clinical remission in ulcerative colitis: a systematic review and meta-analysis. BMJ Open.

[12] Matsuoka K, Kobayashi T, Ueno F (2018). Evidence-based clinical practice guidelines for inflammatory bowel disease. J Gastroenterol.

[13] Yamamoto T, Umegae S, Matsumoto K (2011). Daily granulocyte and monocyte adsorptive apheresis in patients with active ulcerative colitis: a prospective safety and feasibility study. J Gastroenterol.

[14] Kanke K, Nakano M, Hiraishi H, Terano A (2004). Clinical evaluation of granulocyte/monocyte apheresis therapy for active ulcerative colitis. Dig Liver Dis.

[15] Yoshimura N, Yoshimoto H, Yamaka T, Takazoe M (2011). Optimizing GMA therapy for patients with ulcerative colitis-moving towards a more effective GMA regimen. Jpn J Apher.

[16] Habermalz B, Sauerland S (2010). Clinical effectiveness of selective granulocyte, monocyte adsorptive apheresis with the Adacolumn device in ulcerative colitis. Dig Dis Sci.

[17] Suzuki Y, Yoshimura N (2005). The efficacy of granulocyte and monocyte adsorptive apheresis (GCAP) for patients with ulcerative colitis: GCAP can be one of the standard therapies for patients with ulcerative colitis. Jpn J Apher.

[18] Yokoyama Y, Kawai M, Fukunaga K (2013). Looking for predictive factors of clinical response to adsorptive granulocyte and monocyte apheresis in patients with ulcerative colitis: markers of response to GMA. BMC Gastroenterol.

[19] Yokoyama Y, Watanabe K, Ito H (2015). Factors associated with treatment outcome, and long-term prognosis of patients with ulcerative colitis undergoing selective depletion of myeloid lineage leucocytes: a prospective multicenter study. Cytotherapy.

[20] Yokoyama Y, Sawada K, Aoyama N (2020). Efficacy of granulocyte and monocyte adsorptive apheresis in patients with inflammatory bowel disease showing lost response to infliximab. J Crohns Colitis.

